# Balancing Benefits and Harms of COVID-19 Vaccines: Lessons from the Ongoing Mass Vaccination Campaign in Lombardy, Italy

**DOI:** 10.3390/vaccines10040623

**Published:** 2022-04-15

**Authors:** Giovanni Corrao, Federico Rea, Matteo Franchi, Danilo Cereda, Antonio Barone, Catia Rosanna Borriello, Giulia Petra Della Valle, Michele Ercolanoni, Jose Jara, Giuseppe Preziosi, Manuel Maffeo, Francesco Mazziotta, Elisabetta Pierini, Francesco Lecis, Pierfrancesco Sanchirico, Francesco Vignali, Olivia Leoni, Ida Fortino, Massimo Galli, Giovanni Pavesi, Guido Bertolaso

**Affiliations:** 1 National Centre for Healthcare Research and Pharmacoepidemiology, University of Milano-Bicocca, 20126 Milan, Italy; federico.rea@unimib.it (F.R.); matteo.franchi@unimib.it (M.F.); 2 Unit of Biostatistics, Epidemiology and Public Health, Department of Statistics and Quantitative Methods, University of Milano-Bicocca, 20126 Milan, Italy; 3 Directorate General for Health, Lombardy Region, 20124 Milan, Italy; danilo_cereda@regione.lombardia.it (D.C.); catia_rosanna_borriello@regione.lombardia.it (C.R.B.); dellavalle_petra_stg@regione.lombardia.it (G.P.D.V.); manuel.maffeo@unimi.it (M.M.); francesco.mazziotta@unimi.it (F.M.); elisabetta.pierini@unimi.it (E.P.); olivia_leoni@regione.lombardia.it (O.L.); ida_fortino@regione.lombardia.it (I.F.); giovanni_pavesi@regione.lombardia.it (G.P.); 4 ARIA S.p.a., 20124 Milan, Italy; antonio.barone@ariaspa.it (A.B.); michele.ercolanoni@ariaspa.it (M.E.); jose.jara@ext.ariaspa.it (J.J.); giuseppe.preziosi@ariaspa.it (G.P.); 5 EY Advisory, 00187 Rome, Italy; francesco.lecis@it.ey.com (F.L.); pierfrancesco.sanchirico@it.ey.com (P.S.); francesco.vignali@it.ey.com (F.V.); 6 Infectious Diseases Unit, Luigi Sacco Hospital, 20157 Milan, Italy; massimo.galli@unimi.it; 7 Department of Biomedical and Clinical Sciences, University of Milan, 20157 Milan, Italy; 8 Management of the Vaccination Campaign, Lombardy Region, 20124 Milan, Italy; bertolaso1@gmail.com

**Keywords:** COVID-19, healthcare utilization database, venous thromboembolism, effectiveness

## Abstract

**Background**. Limited evidence exists on the balance between the benefits and harms of the COVID-19 vaccines. The aim of this study is to compare the benefits and safety of mRNA-based (Pfizer-BioNTech and Moderna) and adenovirus-vectored (Oxford-AstraZeneca) vaccines in subpopulations defined by age and sex. **Methods**. All citizens who are newly vaccinated from 27 December 2020 to 3 May 2021 are matched to unvaccinated controls according to age, sex, and vaccination date. Study outcomes include the events that are expected to be avoided by vaccination (i.e., hospitalization and death from COVID-19) and those that might be increased after vaccine inoculation (i.e., venous thromboembolism). The incidence rate ratios (IRR) of vaccinated and unvaccinated citizens are separately estimated within strata of sex, age category and vaccine type. When suitable, number needed to treat (NNT) and number needed to harm (NNH) are calculated to evaluate the balance between the benefits and harm of vaccines within each sex and age category. **Results**. In total, 2,351,883 citizens are included because they received at least one dose of vaccine (755,557 Oxford-AstraZeneca and 1,596,326 Pfizer/Moderna). A reduced incidence of COVID-19-related outcomes is observed with a lowered incidence rate ranging from 55% to 89% and NNT values ranging from 296 to 3977. Evidence of an augmented incidence of harm-related outcomes is observed only for women aged <50 years within 28 days after Oxford-AstraZeneca (being the corresponding adjusted IRR of 2.4, 95% CI 1.1–5.6, and NNH value of 23,207, 95% CI 10,274–89,707). **Conclusions**. A favourable balance between benefits and harms is observed in the current study, even among younger women who received Oxford-AstraZeneca.

## 1. Introduction

Huge efforts directed at the development of efficacious and safe vaccines against severe acute respiratory syndrome coronavirus 2 (SARS-CoV-2) have been made by the scientific community and the pharmaceutical industry, backed by government support [1]. In the first phase of the vaccination campaign, the following four vaccines were approved for use against coronavirus disease 2019 (COVID-19) in the European Union: two mRNA-based vaccines (manufactured by Pfizer-BioNTech and Moderna) and two vaccines based on an adenovirus vector (manufactured by Oxford-AstraZeneca and Janssen) [2]. Their capacity to prevent symptomatic COVID-19 has been consistently demonstrated [3,4,5,6,7,8,9].

Concerns over the safety of vaccines have been spreading lately, particularly with respect to adenovirus-vectored vaccines. By mid-March 2021, vaccination with the Oxford-AstraZeneca vaccine ChAdOx1-S was paused in a number of European countries due to reports of thromboembolic events [10]. On 11 March 2021 the European Medicines Agency (EMA) stated that ‘The number of thromboembolic events in vaccinated people is no higher than the number seen in the general population’ [11]. On 23 April 2021 the EMA’s Human Medicines Committee came to the conclusion that very rare blood clots may occur after the administration of adenoviral vector vaccines, mostly in people <60 years of age and with a preponderance in females. Because in countries with low infection rates few direct clinical benefits are derived from vaccinating younger citizens, this observation justified the preferential indication to use adenoviral vector vaccines in individuals aged ≥60 years [12]. Increased rates of venous thromboembolism among people aged 18–65 years within 28 days of vaccination with ChAdOx1-S have recently been reported [13]. Notably, however, the authors of those reports have emphasized that the absolute risks were small and should be interpreted in the context of the benefits of COVID-19 vaccination.

In the current study, the platform specifically designed for monitoring and assessing the vaccination plan in the Italian Lombardy Region was used to compare the benefits and safety of the aforementioned mRNA-based COVID-19 vaccines (Pfizer-BioNTech and Moderna) and an adenovirus-vectored COVID-19 vaccine (Oxford-AstraZeneca) in subpopulations defined by age and sex.

## 2. Methods

### 2.1. Target Population and Data Sources

Residents in Lombardy born before 31 December 2005 (i.e., those who had celebrated their 16th birthday by 27 December 2020—which was the date the vaccination campaign started in Italy—or those who would do so in the course of 2021) formed the target population and consisted of just over 8.7 million candidates for vaccination. Three population-based data sources collecting individual health information were used for carrying out the current study. One, the healthcare utilization database aimed to facilitate Regional Health Service (RHS) management and collects a variety of information including hospital discharge records supplied by public or private hospitals (primary diagnosis, co-existing conditions, and performed procedures coded according to the ICD-9-CM classification system), and outpatient dispensation of drugs reimbursed to pharmacies after filing doctors’ prescriptions (coded according to the Anatomical Therapeutic Chemical (ATC) classification system). More details on the Italian healthcare utilization databases are reported elsewhere [14]. Two, the registry of patients with a confirmed diagnosis of SARS-CoV-2 infection, which was established since 21 February 2020 (i.e., on the date of the first ascertained diagnosis in Lombardy) with the aim of monitoring ascertained infections of SARS-CoV-2 and hospital admissions, emergency room accesses, and deaths due to COVID-19. Diagnoses of COVID-19 were revealed to the Regional Health Authority from the following several sources: public and private hospitals, general practitioners, municipal registries, and laboratories accredited by the Regional Health Authority. Three, the COVID-19 vaccination registry, which was established with the aim of monitoring and evaluating the vaccine campaign since its inception and collects information about date, type, and dose of each dispensed vaccine.

All the different data pertaining to a given individual may be interconnected because a single individual identification code is used by all databases. To preserve privacy, each identification code was automatically converted into an anonymous code. Further details of the healthcare databases used in the context of COVID-19 in Lombardy have been reported previously [15].

According to the rules from the Italian Medicines Agency, retrospective studies using administrative databases do not require Ethics Committee protocol approval. Informed consent was not required since this study involves an administrative database and does not contain any identifiable information that could be linked to any specific participant. All the methods adhered to relevant ethical guidelines for handling human data.

### 2.2. The Italian Vaccination Campaign

The Italian vaccination program started on 27 December 2020 and was structured into the following phases: (i) The following three categories were identified to be vaccinated in the initial phase: health workers, people resident in a nursing home, and people aged 80 years and older. The vaccination campaign for these categories mainly took place during the first two months of 2021, and the mRNA-based (Pfizer-BioNTech or Moderna) vaccines were adopted. (ii) Individuals involved in specific job categories (i.e., school and university staff, armed forces of police, penitentiaries) were prioritized to receive the vaccine because they were in essential categories and/or at high risk for infection. This stage started in March 2021, and the Oxford-AstraZeneca was used. (iii) From April 2021, the vaccination campaign for the general Italian population up to 79 years of age started and was based in a descending fashion on age alone (70–79, 60–69, and <60) as well as on individually listed conditions or diseases that have shown a greater prevalence of severe or lethal COVID-19 in clinical studies. During the study follow-up (up to 3 May 2021), individuals aged 70 years or older and frail people received at least the first dose of the mRNA-based (Pfizer-BioNTech or Moderna) vaccines.

Because the vaccination program plan of the Italian Ministry of Health was based on the best evidence available at that time (March 2021), the mRNA-based vaccines were adopted for individuals at higher risk of developing a severe manifestation of COVID-19.

### 2.3. Cohort Selection and Typing

Among all candidates for vaccination, those who received their first dose of Pfizer-BioNTech, Moderna, or Oxford-AstraZeneca vaccines until 3 May 2021 were identified. Citizens who were not beneficiaries of the RHS, or who became beneficiaries after 1 January 2019 were excluded. The remaining citizens constituted the vaccinated cohort members, the date, and type of vaccine administered were recorded for each.

Whenever a citizen who was vaccinated on a given day (the index date) was identified, from 1 to 10 controls were randomly selected from the remaining vaccination candidates who (i) had not yet been vaccinated, (ii) were the same age and sex as the vaccinated cohort member, and (iii) had been an RHS beneficiary since at least since 1 January 2019. That so-formed cohort constituted the (initially) unvaccinated controls. The formal justification for choosing the number of controls matched to vaccines is provided in the Supplementary Materials Section S1.

Vaccines and controls were typed by recording their age and sex at the index date. Hospital admissions and drug prescriptions experienced within 2 years before the index date were used to investigate a list of 62 conditions possibly affecting the risks of severe/fatal clinical manifestations of SARS-CoV-2 infection, and/or venous thromboembolism [16,17,18]. The total number of contacts that each vaccinated and control had with RHS healthcare services within 2 years before the index date was also recorded. A complete list of these conditions along with the ICD-9 and ATC codes used to track them is provided in Supplementary Table S1.

### 2.4. Measuring Vaccine Benefits

**Figure 1:**
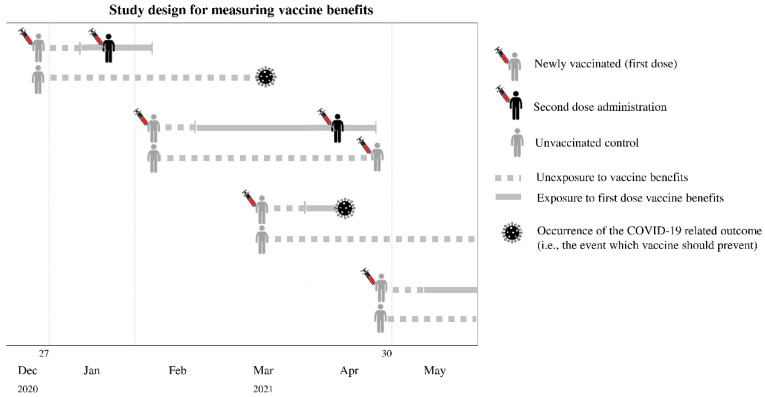
Study design for measuring vaccine benefits.

Each vaccinated citizen was matched 1:1 with an unvaccinated control (Figure 1). Observational person-months started on the index date until the earliest of the following events: outcome occurrence (see below), emigration, death unrelated to COVID-19, or the end of the study period (31 May 2021). Whenever an unvaccinated control was subsequently vaccinated on a given date, follow-up was interrupted at that date for both the unvaccinated control and the corresponding vaccinated cohort member. Person-months were partitioned into subperiods categorised as unexposed, exposed to the first dose, and exposed to both doses of vaccine. The entire follow-up period was categorised as unexposed to unvaccinated controls. Under the assumption that immune coverage is achieved 2 weeks after receiving the vaccine [9], the time-window between the index date and 14 days later was categorized as unexposed for vaccinated cohort members. Exposure to the first dose included the time-window between 14 days after receiving the first dose and the earliest among 14 days after the second dose or the end of follow-up. The period categorized as ‘exposed to the second dose’ started 14 days after receiving the second dose and finished at the end of follow-up. Because few citizens had received the second dose of Oxford-AstraZeneca vaccine by 3 May 2021, observations were censured at the date corresponding to 14 days after the second dose of vaccine was administered (i.e., only the benefits due to the first dose were assessed).

A composite outcome was built comprising hospital admission for COVID-19, admission to an intensive care unit for COVID-19, and death from COVID-19. A patient was considered to have experienced the outcome if at least one of these events occurred during the observation period, and the earliest event was considered the date of its occurrence.

### 2.5. Measuring Vaccine Harm

**Figure 2:**
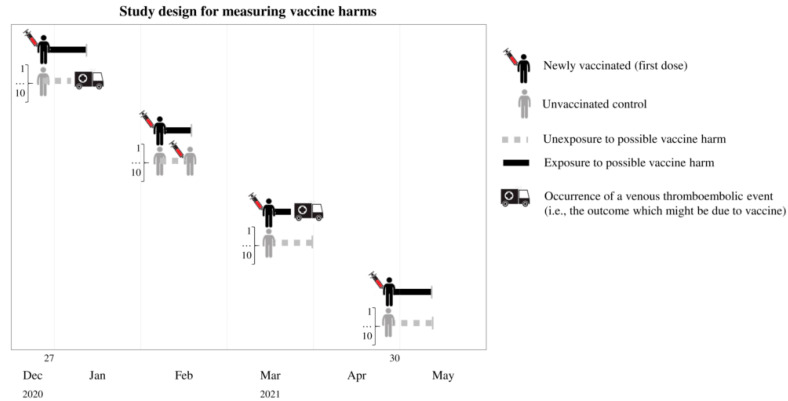
Study design for measuring vaccine harms.

Vaccinated citizens were matched to unvaccinated controls at a 1:10 ratio (Figure 2). Each cohort member accumulated person-months of observation starting on the index date until the earliest of the following events: outcome occurrence (see below), emigration, death from any cause, or 28 days after the index date. Follow-up was interrupted for all the members of a given 1:10 risk-set at the earliest date when an unvaccinated control was vaccinated.

Person-months of exposure and non-exposure, respectively, started at the date of vaccination and the corresponding date for unvaccinated controls. The outcomes of interest included admission to hospital or an emergency room with a primary or secondary diagnosis of venous thromboembolism. The list of conditions used to track venous thromboembolism was the same as that used in a recently reported investigation [19], and the corresponding ICD-9 codes are given in Supplementary Table S2.

### 2.6. Main Analyses

Standardized mean differences were calculated to compare baseline characteristics in vaccinated subjects and controls. In accordance with Austin [20], standardized mean differences <0.10 were considered negligible. Subsequent analyses were performed separately for sex, five age categories recorded at the index date (<50, 50–59, 60–69, 70–79, and ≥80 years), and type of vaccine administered; adenovirus-vectored (Oxford-AstraZeneca) or mRNA-based (Pfizer-BioNTech or Moderna). Incidence rates were compared by the mean incidence rate ratios (IRRs) and 95% confidence intervals (CIs), with the latter estimated via normal approximation. Both unadjusted and adjusted IRR estimates were obtained using Poisson regression modelling. With respect to IRR adjustment, of the 62 typing conditions only those to which at least 1% of the vaccinated cohort were exposed were included.

Difference metric, as opposed to IRR, was used to compare subperiod-specific incidence rates. This was performed using the number needed to treat (NNT) to measure vaccine benefits and the number needed to harm (NNH) to measure vaccine-associated harm. A low NNT value indicates more favourable effects of vaccination, because few citizens would need to be treated to expect one citizen to benefit. Similarly, NNH assesses undesirable treatment outcomes by estimating the number of citizens vaccinated per one harmful event. Therefore, a desirable vaccination profile would have a low NNT and a high NNH [21].

NNT and NNH were calculated only in the presence of significant evidence of both protective (avoiding severe/fatal clinical manifestation of SARS-CoV-2 infection) and harmful action (increasing venous thromboembolic events) [22]. Where suitable, NNT and NNH were calculated directly as the reciprocal of the absolute difference in incidence rates, with the corresponding 95% CI calculated as the inverse of the CI around the absolute incidence rate difference. Adjusted NNT, adjusted NNH, and corresponding 95% CIs were indirectly calculated from the adjusted IRR estimates [23].

### 2.7. Supplementary Analyses

A range of prespecified supplementary analyses were conducted.

One, the first occurring among specific events used to measure vaccine benefits (i.e., hospital admission, admission to an emergency room, and death with diagnosis of COVID-19), and vaccine harms (cerebral venous thrombosis and other venous thrombosis), were calculated separately.

Two, due to the arbitrary nature of the choice of the time-window length after vaccination during which no exposure to vaccine benefits was assumed because immunity was gradually building, as well as the 14-day window used in the main analysis a shorter period (7 days) and a longer period (21 days) were considered.

Three, the choice of the time-window length with respect to the risk of developing venous thromboembolism was also arbitrary, so as well as the 28-day window used in the main analysis a shorter period (21 days) and a longer period (35 days) were considered.

Four, as well as multiple adjustments conducted by including covariates in the models, an approach based on propensity scores was utilized [24]. Propensity scores—i.e., individualized probabilities of receiving a vaccine based on the above-described set of 62 conditions for typing vaccinated subjects and controls—were generated by fitting a Cox proportional hazards model including the above covariates as predictors and time to vaccination as the outcome. Propensity score-adjusted estimates were then generated.

For all hypotheses tested, two-tailed *p* values <0.05 were considered significant.

## 3. Results

### 3.1. Study Population

Of the 2,381,883 beneficiaries of the RHS who were vaccinated by 3 May 2021, almost a third (31.1%) received the Oxford-AstraZeneca vaccine. There was a lower prevalence of diabetes among subjects vaccinated with Oxford-AstraZeneca, and a higher prevalence of dyslipidaemia among subjects vaccinated with mRNA-based vaccines (Table 1). More frequent contacts with RHS services were recorded among vaccinated subjects than among unvaccinated controls.

### 3.2. Benefit-Related Outcomes

During a median follow-up of 35 days (interquartile range: 12–37 days), 639 clinically severe infections were documented among vaccinated subjects and 6921 were documented among controls, equating to respective numbers of cases per 10,000 person-months of 4.8 and 16.9. In vaccinated subjects, the respective incidence rates of hospital admission, emergency room access, and mortality were 4.52, 0.03, and 0.28 per 10,000 person-months, and in unvaccinated subjects they were 16.03, 0.18, and 0.64.

### 3.3. Harm-Related Outcomes

During a median follow-up of 28 days (interquartile range: 12–28 days), 307 venous thromboembolic events were documented among vaccinated subjects (1.8 cases per 10,000 person-months) and 4327 were documented among control subjects (2.7 cases per 10,000 person-months). The respective incidence rates of cerebral venous thrombosis and other venous thrombosis were 0.02 and 1.86 per 10,000 person-months spent during exposure periods, and 0.01 and 2.67 per 10,000 person-months spent during unexposed periods.

### 3.4. Benefits and Harms Associated with Oxford-AstraZeneca Vaccination

There was evidence of reduced COVID-19-related outcomes until the age of 79 years, with adjusted IRRs decreasing from 69% (95% CI 44–83%) in women aged 60–69 years, to 89% (95% CI 77–95%) in women aged <50 years, and from 72% (95% CI 57–82%) in men aged 70–79 years, to 85% (95% CI 70–92%) in men aged 50–59 years (Table 2). NNT values ranged from 679 (222–860) in men aged 70–79 years, to 2796 (319–4283) in men aged <50 years, and from 1209 (177–1550) in women aged 70–79 years, to 3063 (210–3957) in women aged <50 years. Evidence of increased harm-related outcomes was only observed in women aged <50 years, with an adjusted IRR of 2.43 (95% CI 1.05–5.63) and a NNH value of 23,207 (10,274–89,707).

### 3.5. Benefits and Harms Associated with Pfizer or Moderna Vaccination

There was evidence of a reduced incidence of COVID-19-related outcomes associated with Pfizer or Moderna vaccination, with adjusted lowering IRR ranging from 55% (95% CI 48–60%) in women aged ≥80 years, to 86% (69–94%) in women aged 50–59 years, and from 59% (52–65%) in men aged ≥80 years, to 82% (64–91%) in men aged 50–59 years (Table 3). In women the NNT values ranged from 463 (411–529) in the youngest age group to 3479 (2678–4964) in the oldest age group. In men the NNT values ranged from 296 (262–339) in the youngest age group to 3977 (2777–7002) in the oldest age group. There was a weak increase in harm-related outcomes in women in the youngest age group, with an unadjusted IRR of 2.62 (95% CI 1.31–5.26) and an NNH value of 100,291, but these results were not statistically significant after estimates adjustment.

### 3.6. Supplementary Analyses

IRR estimates were generally similar to those obtained in the main analyses, irrespective of the time-window length from non-exposure to benefit after vaccination (Table S3). They were also similar with respect to the development of venous thromboembolism (Table S4). The propensity score-adjusted estimates were similar to those obtained via conventional adjustment (Table S5), with the exception of increased harm-related outcomes in women in the youngest age group vaccinated with Pfizer or Moderna.

## 4. Discussion

The benefits of the COVID-19 vaccination campaign estimated in the current study must be interpreted with reference to previous observations. The effectiveness of vaccination for preventing severe forms of infection observed in the study is consistent with that expected based on previously reported randomized clinical trials and observational studies [3,4,5,6,7,8,9]. Relative reductions after the first dose ranged from 55% in women aged ≥80 years who received mRNA-based vaccines to 89% in women aged <50 years who received Oxford-AstraZeneca.

The effectiveness of the vaccination campaign with respect to avoiding outcomes that would have occurred in the absence of vaccination clearly depends on vaccine efficacy, but it also depends on both the speed of the vaccination campaign and the types of people vaccinated. The more citizens that are vaccinated, the greater the expected benefits will be, particularly if more people vulnerable to severe clinical manifestations of infection are vaccinated. During the first months from the start of the vaccination campaign in Lombardy, the incidence rates were strongly reduced in older people. From 13 to 33 cases every 10,000 person-months were avoided among citizens aged ≥80 years, but only 2 or 3 cases every 10,000 person-months were avoided among younger citizens. This leads to the obvious consideration that the clinical impact of the campaign is expected to be much higher in older citizens, and in general, in clinically more vulnerable citizens. The current study was limited to estimating clinical benefits, and among these, those applying to more severe symptomatic COVID-19 cases. The effects of the vaccination campaign on asymptomatic SARS-CoV-2 infections, paucisymptomatic COVID-19 disease, and the progressive reduction of viral spread were not investigated.

In the current study, there was an increased rate of venous thromboembolic events 28 days after vaccination with Oxford-AstraZeneca in women aged <50 years. Consistently, there was an almost doubled rate 28 days after the Oxford-AstraZeneca vaccination in recipients aged 18–65 years in Denmark and Norway, and an even greater increase in younger citizens and in women [13]. In the present study, there was a weak signal that younger women may be at an increased risk of venous thromboembolic events after the administration of mRNA-based vaccines, but the association was not statistically significant after adjustment for covariates. Notably, the size of the relevant age subgroup was small.

The present study provides evidence that the benefits of the Oxford-AstraZeneca vaccination clearly outweigh the corresponding risk of venous thromboembolic events, even in younger women. Vaccination of a little more than 3000 citizens yielded a unit of therapeutic benefit, whereas vaccination of >23,000 was required to yield a harmful event (a ratio of approximately 7.6-fold). Therefore, these findings support future vaccination programs in low- and middle-income countries (where the percentage of people vaccinated is very low) as well as in high-income countries (in which the third and fourth doses are ongoing/planned) through two main policy directions. First, the better benefit-harm profile of mRNA-based vaccines should be considered for managing vaccination campaigns in younger women. Second, the Oxford-AstraZeneca vaccine should be considered for younger women in the absence of the mRNA-based vaccines since the benefits outweigh the risks, at least with the virus spread observed during the study period (see below for a specific comment on this issue).

Four further issues pertaining to interpreting the results of the current study should be discussed. First, few citizens had received the second dose of the Oxford-AstraZeneca vaccine by the endpoint of the study because it was scheduled for 12 weeks after the first one; therefore, we only assessed clinical benefits after the first dose. Because additional cases of severe/fatal infection are expected to be avoided after the second dose; however, the benefit-harm profile is expected to improve as more citizens complete the scheduled vaccination cycle [9]. Second, the relatively high rates of COVID-19 infection during the investigation period clearly affected our estimates. For example, there was an incidence rate of 3.7 cases of severe/fatal clinical manifestations of COVID-19 every 10,000 person-months accumulated by unvaccinated women aged <50 years who received Oxford-AstraZeneca. It was calculated that the incidence rate should fall to 0.46 cases per 10,000 person-months in order to equalise NNT and NNH, which is to make the benefit equal to the harm. Third, the possible resistance of SARS-CoV-2 variants to COVID-19 vaccines raises concerns [25,26]. Rapid diffusion of a variant occurred in the study region of Lombardy during the study follow-up, specifically the B.1.1.7. variant. Its diffusion decreased; however, making way for the sporadic appearance of the P.1. variant and mostly the B.1.167.2 variant, the latter rapidly increasing from May to June 2021 (after the study period) [27]. This implies that our study estimates the average impact of the vaccination campaign over multiple strains, but careful day-by-day monitoring is crucial to track mutations and evaluate their effects on the vaccination campaign. Lastly, we observed that women aged <40 years had incidences of venous thromboembolic events even higher with respect to those observed for women aged <50 years, with the IRR (and 95% CI) respectively associated with Oxford-AstraZeneca and mRNA-vectored vaccines of 4.8 (1.4–15.9) and 2.3 (0.8–6.8). This confirms that vaccine-related thromboembolic events mainly regard younger women.

The main strength of the present study is its population-based approach, implemented in a setting with regional health services providing free access to healthcare and well-defined and near-complete follow-up based on computerised registries with full population coverage and daily updates [9]. The large size of the target population and the large amounts of accumulated person-time with respect to exposure and non-exposure to vaccine harms enabled the study to attain sufficient power to analyse relatively rare events. The profiling of the target population through the clinical ‘footprints’ of real patients as they accessed their routine medical care is also a strength of the study. Lastly, a number of sensitivity analyses confirmed the robustness of the study results.

The present study also had potential weaknesses. First, the validity of the results ultimately depends on the accurate coding of outcomes [13]. There are potential concerns with regard to the diagnosis of venous thromboembolisms with clinical complexity that is not captured completely by any single ICD-9 code. In addition, less serious adverse events may have gone undetected. Second, the two mRNA-based vaccines were analysed together in our study because of the few doses of Moderna used during the study follow-up. However, since the Pfizer-BioNTech and Moderna vaccines showed differences both in the effectiveness and harms [28,29], future research on this topic is needed. Third, because of the lack of evidence from up-to-date studies performed in the healthcare system of the region we studied and because hospital records were not available for scrutiny, misclassification of diagnoses cannot be completely excluded in our setting. Finally, as with any observational study, the present study may have been affected by differences between vaccinated and unvaccinated citizens, particularly in terms of health-seeking behavior. Several attempts were made to take such concerns into account, particularly by adjusting for individual vulnerability factors that encourage the anticipation of vaccine supply and increase the risk of severe/fatal manifestations of SARS-CoV-2 infection. Adjusted estimates were consistent with those obtained via a propensity score approach. The main findings were consistent with those obtained after adjustment for the total amount of previous healthcare received by each vaccinated subject and control, constituting further evidence of exchangeability.

## 5. Conclusions

The evidence of the effectiveness of mRNA-based and adenovirus vector-based vaccines for preventing severe/fatal forms of SARS-CoV-2 infection was obtained in the current large population-based investigation. An increased rate of venous thromboembolism within 28 days after the first dose of Oxford-AstraZeneca in women aged <50 years was observed. The most important observation in the present study was that the balance between benefits and harms was largely skewed towards vaccination, even in younger women. mRNA-based vaccines should be preferred for young women because of the better risk-benefit profile.

## Figures and Tables

**Table 1:** Demographic and clinical characteristics of vaccinated and unvaccinated citizens at baseline. Italy, Lombardy Region, 27 December 2020 to 3 May 2021.

	AstraZeneca			Pfizer or Moderna		
	Vaccinated (N = 755,557)	Unvaccinated (N = 755,557)	SMD	Vaccinated (N = 1,596,326)	Unvaccinated (N = 1,596,326)	SMD
Women						
<50 years	110,500 (24.7%)	110,500 (24.7%)	MV	145,584 (15.7%)	145,584 (15.7%)	MV
50–59 years	57,671 (12.9%)	57,671 (12.9%)		103,873 (11.2%)	103,873 (11.2%)	
60–69 years	80,917 (18.1%)	80,917 (18.1%)		127,078 (13.7%)	127,078 (13.7%)	
70–79 years	195,128 (43.6%)	195,128 (43.6%)		153,191 (16.6%)	153,191 (16.6%)	
≥80 years	2941 (0.7%)	2941 (0.7%)		394,754 (42.7%)	394,754 (42.7%)	
Men						
<50 years	55,659 (18.0%)	55,659 (18.0%)	MV	89,336 (13.3%)	89,336 (13.3%)	MV
50–59 years	27,181 (8.8%)	27,181 (8.8%)		65,951 (9.8%)	65,951 (9.8%)	
60–69 years	65,065 (21.1%)	65,065 (21.1%)		120,553 (17.9%)	120,553 (17.9%)	
70–79 years	158,639 (51.4%)	158,639 (51.4%)		146,410 (21.8%)	146,410 (21.8%)	
≥80 years	1856 (0.6%)	1856 (0.6%)		249,596 (37.1%)	249,596 (37.1%)	
Risk factors for SARS-CoV-2 severe clinical manifestations						
Solid malignancies and neoplasm of lymphatic and haematopoietic tissue	15,972 (2.1%)	24,084 (3.2%)	−0.067	91,288 (5.7%)	63,503 (4.0%)	0.081
Hypothyroidism	41,279 (5.5%)	43,637 (5.8%)	−0.014	119,161 (7.5%)	100,077 (6.3%)	0.047
Diabetes without insulin therapy	35,120 (4.7%)	53,418 (7.1%)	−0.103	169,941 (10.7%)	134,946 (8.5%)	0.075
Insulin therapy	2484 (0.3%)	15,330 (2.0%)	−0.158	66,335 (4.2%)	43,289 (2.7%)	0.079
Dyslipidaemia	167,512 (22.2%)	165,865 (22.0%)	0.005	495,679 (31.1%)	403,997 (25.3%)	0.128
Gout	23,052 (3.1%)	28,043 (3.7%)	−0.037	118,534 (7.4%)	100,062 (6.3%)	0.046
Autoimmune haemolytic anaemias, other anaemias, anaemias only tracked from drug therapy	48,496 (6.4%)	61,673 (8.2%)	−0.067	227,932 (14.3%)	199,795 (12.5%)	0.052
Psychosis	10,039 (1.3%)	15,095 (2.0%)	−0.052	65,713 (4.1%)	61,796 (3.9%)	0.013
Depression	73,365 (9.7%)	72,610 (9.6%)	0.003	236,767 (14.8%)	204,808 (12.8%)	0.058
Parkinson’s disease and parkinsonism	5373 (0.7%)	7439 (1.0%)	−0.030	32,637 (2.0%)	29,834 (1.9%)	0.013
Epilepsy and recurrent seizures	8810 (1.2%)	13,748 (1.8%)	−0.054	47,753 (3.0%)	37,401 (2.3%)	0.040
Glaucoma	23,734 (3.1%)	21,864 (2.9%)	0.014	78,886 (4.9%)	69,309 (4.3%)	0.029
Ischaemic heart disease/angina	10,882 (1.4%)	15,155 (2.0%)	−0.043	68,664 (4.3%)	59,692 (3.7%)	0.029
Arrhythmia	20,037 (2.7%)	21,561 (2.9%)	−0.012	96,275 (6.0%)	80,751 (5.1%)	0.043
Hypertension	140,756 (18.6%)	137,931 (18.3%)	0.010	430,070 (26.9%)	373,414 (23.4%)	0.082
Coronary and peripheral vascular disease	54,103 (7.2%)	59,040 (7.8%)	−0.025	186,319 (11.7%)	160,611 (10.1%)	0.052
Oral anticoagulant agents	22,695 (3.0%)	28,929 (3.8%)	−0.045	148,215 (9.3%)	129,640 (8.1%)	0.041
Other diseases of the circulatory system	10,506 (1.4%)	14,921 (2.0%)	−0.045	56,681 (3.6%)	45,482 (2.9%)	0.040
Chronic obstructive pulmonary disease, asthma, chronic respiratory disease only tracked from drug therapy	67,906 (9.0%)	66,118 (8.8%)	0.008	170,732 (10.7%)	152,844 (9.6%)	0.037
Other diseases of the respiratory system	5695 (0.8%)	9586 (1.3%)	−0.051	39,612 (2.5%)	36,213 (2.3%)	0.014
Inflammatory bowel diseases (Ulcerative colitis and Chron’s disease)	13,349 (1.8%)	13,227 (1.8%)	0.001	38,463 (2.4%)	29,252 (1.8%)	0.040
Other diseases of the digestive system	16,503 (2.2%)	17,227 (2.3%)	−0.006	52,822 (3.3%)	45,202 (2.8%)	0.028
Other diseases of the genitourinary system	13,881 (1.8%)	14,790 (2.0%)	−0.009	44,839 (2.8%)	40,361 (2.5%)	0.017
Diseases of the skin and subcutaneous tissues, including no rheumatoid psoriasis	9347 (1.2%)	10,722 (1.4%)	−0.016	27,481 (1.7%)	23,801 (1.5%)	0.018
Other diseases of the musculoskeletal system and connective tissue	17,137 (2.3%)	16,166 (2.1%)	0.009	43,326 (2.7%)	36,357 (2.3%)	0.028
Symptoms, signs, and ill-defined conditions	4700 (0.6%)	7611 (1.0%)	−0.043	30,252 (1.9%)	25,403 (1.6%)	0.023
Chronic pain	21,321 (2.8%)	24,775 (3.3%)	−0.027	93,131 (5.8%)	81,542 (5.1%)	0.032
Corticosteroids	80,619 (10.7%)	85,663 (11.3%)	−0.021	231,005 (14.5%)	195,454 (12.2%)	0.065
Hormone therapy (oral contraceptives or replacement hormone therapy) among women	10,841 (2.4%)	9090 (2.0%)	0.020	17,686 (1.9%)	14,931 (1.6%)	0.017
Number of contacts with healthcare services of RHS						
<5	273,563 (36.2%)	253,203 (33.5%)	0.057	333,773 (20.9%)	473,336 (29.7%)	−0.202
5–100	400,607 (53.0%)	445,051 (58.9%)	−0.119	901,350 (56.5%)	836,878 (52.4%)	0.081
>100	81,387 (10.8%)	57,303 (7.6%)	0.111	361,203 (22.6%)	286,112 (17.9%)	0.117

SMD: standardized mean difference; MV: matching variable.

**Table 2:** Benefits—harms profile of Oxford-AstraZeneca vaccine (first dose) in women and men of five age categories.

Women									
Age Category (Years)	Exposure to Vaccine Benefits (First Dose)			Unexposure to Vaccine Benefits			Incidence Rate Ratio (95% CI) ^(a)^		Number Needed to Treat (95% CI) ^(b)^
	Events	PM	IR (per 10,000 PM)	Events	PM	IR (per 10,000 PM)	Unadjusted	Adjusted	
<50	7	198,035	0.35	111	302,575	3.67	0.10 (0.05 to 0.22)	0.11 (0.05 to 0.23)	3063 (210 to 3957)
50–59	8	95,952	0.83	78	149,102	5.23	0.16 (0.08 to 0.33)	0.16 (0.08 to 0.34)	2274 (180 to 3226)
60–69	12	52,563	2.28	100	117,698	8.50	0.27 (0.15 to 0.49)	0.31 (0.17 to 0.56)	1609 (241 to 2472)
70–79	10	58,529	1.71	190	190,324	9.98	0.17 (0.09 to 0.32)	0.20 (0.10 to 0.38)	1209 (177 to 1550)
≥80	1	1997	5.01	8	4373	18.29	0.27 (0.03 to 2.19)	0.30 (0.04 to 2.49)	-
Total	38	407,077	0.93	487	764,072	6.37			
**Age Category (Years)**	**Exposure to Vaccine Harms (First Dose)**			**Unexposure To Vaccine Harms**			**Incidence Rate Ratio (95% CI) ^(a)^**		**Number Needed to Harm (95% CI) ^(b)^**
	**Events**	**PM**	**IR (per 10,000 PM)**	**Events**	**PM**	**IR (per 10,000 PM)**	**Unadjusted**	**Adjusted**	
<50	7	99,556	0.70	30	995,563	0.30	2.33 (1.02 to 5.31)	2.43 (1.05 to 5.63)	23,207 (10,274 to 89,707)
50–59	4	51,391	0.78	28	513,884	0.54	1.43 (0.50 to 4.07)	1.53 (0.54 to 4.38)	-
60–69	4	56,267	0.71	53	562,560	0.94	0.75 (0.27 to 2.08)	0.78 (0.28 to 2.18)	-
70–79	12	100,237	1.20	293	1,001,500	2.93	0.41 (0.23 to 0.73)	0.39 (0.21 to 0.72)	-
≥80	0	2191	0.00	12	21,792	5.51	-	-	-
Total	27	309,642	0.87	416	3,095,299	1.34			
**Men**									
**Age Category (Years)**	**Exposure to Vaccine Benefits (First Dose)**			**Unexposure to Vaccine Benefits**			**Incidence Rate Ratio (95% CI) ^(a)^**		**Number Needed to Treat (95% CI) ^(b)^**
	**Events**	**PM**	**IR (per 10,000 PM)**	**Events**	**PM**	**IR (per 10,000 PM)**	**Unadjusted**	**Adjusted**	
<50	11	107,608	1.02	74	160,921	4.60	0.22 (0.15 to 0.48)	0.27 (0.15 to 0.49)	2796 (319 to 4283)
50–59	8	49,469	1.62	90	75,166	11.97	0.14 (0.08 to 0.30)	0.15 (0.08 to 0.30)	966 (123 to 1313)
60–69	9	38,341	2.35	112	89,850	12.47	0.19 (0.09 to 0.37)	0.20 (0.10 to 0.39)	988 (133 to 1366)
70–79	22	45,579	4.83	296	151,433	19.55	0.25 (0.16 to 0.38)	0.28 (0.18 to 0.43)	679 (222 to 860)
≥80	2	1277	15.66	10	2772	36.08	0.43 (0.10 to 1.98)	0.46 (0.09 to 2.25)	-
Total	52	242,274	2.15	582	480,142	12.12			
**Age Category (Years)**	**Exposure to Vaccine Harms (First Dose)**			**Unexposure To Vaccine Harms**			**Incidence Rate Ratio (95% CI) (a)**		**Number Needed to Harm (95% CI) (b)**
	**Events**	**PM**	**IR (per 10,000 PM)**	**Events**	**PM**	**IR (per 10,000 PM)**	**Unadjusted**	**Adjusted**	
<50	1	50,669	0.20	33	506,677	0.65	0.30 (0.04–2.22)	0.29 (0.04–2.12)	-
50–59	3	24,608	1.22	28	246,071	1.14	1.07 (0.33–3.52)	1.12 (0.34–3.69)	-
60–69	8	43,846	1.82	98	438,323	2.24	0.82 (0.40–1.68)	0.81 (0.39–1.68)	-
70–79	14	79,934	1.75	332	797,823	4.16	0.42 (0.25–0.72)	0.40 (0.24–0.69)	-
≥80	1	1374	7.28	10	13,623	7.34	0.99 (0.13–7.75)	1.18 (0.14–9.88)	-
Total	27	200,431	1.35	501	2,002,517	2.50			

PM: Person-Months; IR: Incidence Rate. ^(a)^ Incidence Rate Ratio (and 95% Confidence Interval) based on Poisson Regression Model. Models were unadjusted and adjusted for covariate listed in supplementary Table S1. ^(b)^ Number needed to treat and number needed to harm (and corresponding 95% Confidence Interval) were estimated provided that significant benefits and risks were respectively observed from the corresponding Incidence Rate Ratios.

**Table 3:** Benefits—harms profile of mRNA-based vaccines (Pfizer or Moderna) in women and men of five age categories.

Women									
Age Category (Years)	Exposure to Vaccine Benefits (First Dose)			Unexposure to Vaccine Benefits			Incidence Rate Ratio (95% CI) ^(a)^		Number Needed to Treat (95% CI) ^(b)^
	Events	PM	IR (per 10,000 PM)	Events	PM	IR (per 10,000 PM)	Unadjusted	Adjusted	
<50	13	100,665	1.29	196	490,974	3.99	0.32 (0.19 to 0.57)	0.28 (0.16 to 0.50)	3479 (2678 to 4964)
50–59	6	67,909	0.88	180	318,755	5.65	0.16 (0.07 to 0.35)	0.14 (0.06 to 0.31)	2059 (1690 to 2635)
60–69	9	48,052	1.87	178	202,892	8.77	0.21 (0.11 to 0.43)	0.20 (0.10 to 0.39)	1425 (1140 to 1898)
70–79	20	45,489	4.40	234	164,078	14.26	0.31 (0.21 to 0.52)	0.29 (0.18 to 0.46)	988 (785 to 1331)
≥80	248	142,079	17.46	2248	572,002	39.30	0.44 (0.40 to 0.53)	0.45 (0.40 to 0.52)	463 (411 to 529)
Total	296	404,194	7.32	3036	1,748,702	17,36			
**Age Category (Years)**	**Exposure to Vaccine Harms (First Dose)**			**Unexposure To Vaccine Harms**			**Incidence Rate Ratio (95% CI) ^(a)^**		**Number Needed to Harm (95% CI) ^(b)^**
	**Events**	**PM**	**IR (per 10,000 PM)**	**Events**	**PM**	**IR (per 10,000 PM)**	**Unadjusted**	**Adjusted**	
<50	10	131,525	0.76	38	1,309,922	0.29	2.62 (1.31 to 5.26)	1.65 (0.79 to 3.47)	-
50–59	2	91,961	0.22	34	916,009	0.37	0.59 (0.14 to 2.44)	0.40 (0.09 to 1.67)	-
60–69	10	84,627	1.18	93	843,316	1.10	1.07 (0.56 to 2.06)	0.78 (0.40 to 1.50)	-
70–79	16	85,737	1.87	273	854,630	3.19	0.58 (0.35 to 0.97)	0.45 (0.27 to 0.76)	-
≥80	99	268,027	3.69	1385	2,603,444	5.32	0.69 (0.57 to 0.85)	0.70 (0.57 to 0.85)	-
Total	137	661,878	2.07	1823	6,527,321	2.79			
**Men**									
**Age Category (Years)**	**Exposure to Vaccine Benefits (First Dose)**			**Unexposure to Vaccine Benefits**			**Incidence Rate Ratio (95% CI) ^(a)^**		**Number Needed to Treat (95% CI) ^(b)^**
	**Events**	**PM**	**IR (per 10,000 PM)**	**Events**	**PM**	**IR (per 10,000 PM)**	**Unadjusted**	**Adjusted**	
<50	8	61,389	1.30	98	272,808	3.59	0.36 (0.18 to 0.78)	0.30 (0.14 to 0.63)	3977 (2777 to 7002)
50–59	9	42,272	2.13	166	171,452	9.68	0.22 (0.12 to 0.44)	0.18 (0.09 to 0.36)	1260 (1013 to 1666)
60–69	21	44,774	4.69	315	187,785	16.77	0.28 (0.18 to 0.44)	0.24 (0.16 to 0.38)	784 (651 to 988)
70–79	36	42,957	8.38	393	161,173	24.38	0.34 (0.26 to 0.51)	0.33 (0.23 to 0.46)	612 (501 to 786)
≥80	179	76,801	23.31	1844	321,868	57.29	0.41 (0.36 to 0.49)	0.41 (0.35 to 0.48)	296 (262 to 339)
Total	253	268,193		2816	1,115,086				
**Age Category (Years)**	**Exposure to Vaccine Harms (First Dose)**			**Unexposure to Vaccine Harms**			**Incidence Rate Ratio (95% CI) ^(a)^**		**Number Needed to Harm (95% CI) ^(b)^**
	**Events**	**PM**	**IR (per 10,000 PM)**	**Events**	**PM**	**IR (per 10,000 PM)**	**Unadjusted**	**Adjusted**	
<50	1	81,069	0.12	18	806,954	0.22	0.55 (0.07 to 4.14)	0.46 (0.06 to 3.53)	-
50–59	12	58,442	2.05	47	581,519	0.81	2.54 (1.35 to 4.79)	1.69 (0.85 to 3.37)	-
60–69	23	80,154	2.87	175	798,403	2.19	1.31 (0.85 to 2.02)	0.93 (0.59 to 1.45)	-
70–79	22	81,567	2.70	322	812,848	3.96	0.68 (0.44 to 1.05)	0.60 (0.39 to 0.93)	-
≥80	58	158,754	3.65	1025	1,530,971	6.70	0.55 (0.42 to 0.71)	0.53 (0.41 to 0.70)	-
Total	116	459,986		1587	4,530,695				

PM: Person-Months; IR: Incidence Rate. ^(a)^ Incidence rate ratio (and 95% confidence interval) based on Poisson regression model. Models were unadjusted and adjusted for covariates listed in supplementary Table S1 ^(b)^ Number needed to treat and number needed to harm (and corresponding 95% confidence interval) were estimated provided that significant benefits and risks were respectively observed from the corresponding incidence rate ratios.

## Data Availability

The data that support the findings of this study are available from Lombardy Region, but restrictions apply to the availability of these data, which were used under license for the current study, and so are not publicly available. Data are however available from the Lombardy Region upon reasonable request.

## Supplementary Materials

The following supporting information can be downloaded at: https://www.mdpi.com/article/10.3390/vaccines10040623/s1, Table S1: List of conditions used for typifying vaccinated cohort members and unvaccinated controls; Table S2: ICD-9 codes used for tracking venous thromboembolic outcome events; Table S3: Benefit profile according with vaccine type, gender and age category obtained by changing the time-window length after vaccine inoculation assumed of not involving vaccination protection because immunity is gradually building. Time-windows of 7 and 21 days were considered for this secondary analysis alternative to 14 days of the main analysis; Table S4: Harm profile according with vaccine type, gender and age category obtained by changing the time-window length at risk of developing venous thromboembolism. Time-windows of 21 and 35 days were considered for this secondary analysis alternative to 28 days of the main analysis; Table S5: Benefit –harm profiles according with vaccine type, gender and age category obtained by propensity score adjustment. Ref. [30] is cited in Supplementary Materials.

## Code Availability

Statistical analysis was performed using the Statistical Analysis System Software (version 9.4; SAS Institute, Cary, NC, USA). Codes can be obtained by contacting the corresponding author.
