# Supplementary material for: Balancing Benefits and Harms of COVID-19 Vaccines: Lessons from the Ongoing Mass Vaccination Campaign in Lombardy, Italy
Source: Vaccines (Basel). 2022 Apr 15;10(4):623. doi: 10.3390/vaccines10040623 (PMC9028212; doi:10.3390/vaccines10040623)
Supplement: Supplementary file 1 [file vaccines-10-00623-s001.zip › vaccines-1653844-supplementary.pdf]

Supplementary material

Balancing Benefits and Harms of COVID-19 Vaccines: Lessons from the Ongoing Mass Vaccination Campaign in Lombardy, Italy

Giovanni Corrao [1,2,*], Federico Rea [1,2], Matteo Franchi [1,2], Danilo Cereda [3], Antonio Barone [4], Catia Rosanna Borriello [3], Giulia Petra Della Valle [3], Michele Ercolanoni [4], Jose Jara [4], Giuseppe Preziosi [4], Manuel Maffeo [3], Francesco Mazziotta [3], Elisabetta Pierini [3], Francesco Lecis [5], Pierfrancesco Sanchirico [5], Francesco Vignali [5], Olivia Leoni [3], Ida Fortino [3], Massimo Galli [6,7], Giovanni Pavesi [3] and Guido Bertolaso [8]

[1] National Centre for Healthcare Research and Pharmacoepidemiology, University of Milano-Bicocca,
20126 Milan, Italy; federico.rea@unimib.it (F.R.); matteo.franchi@unimib.it (M.F.)

[2] Unit of Biostatistics, Epidemiology and Public Health, Department of Statistics and Quantitative Methods, University of Milano-Bicocca, 20126 Milan, Italy

[3] Directorate General for Health, Lombardy Region, 20124 Milan, Italy; danilo_cereda@regione.lombardia.it (D.C.); catia_rosanna_borriello@regione.lombardia.it (C.R.B.); dellavalle_petra_stg@regione.lombardia.it (G.P.D.V.); manuel.maffeo@unimi.it (M.M.); francesco.mazziotta@unimi.it (F.M.); elisabetta.pierini@unimi.it (E.P.); olivia_leoni@regione.lombardia.it (O.L.); ida_fortino@regione.lombardia.it (I.F.); giovanni_pavesi@regione.lombardia.it (G.P.)

[4] ARIA S.p.a., 20124 Milan, Italy; antonio.barone@ariaspa.it (A.B.); michele.ercolanoni@ariaspa.it (M.E.); jose.jara@ext.ariaspa.it (J.J.); giuseppe.preziosi@ariaspa.it (G.P.)

[5] EY Advisory, 00187 Rome, Italy; francesco.lecis@it.ey.com (F.L.); pierfrancesco.sanchirico@it.ey.com (P.S.); francesco.vignali@it.ey.com (F.V.)

[6] Infectious Diseases Unit, Luigi Sacco Hospital, 20157 Milan, Italy; massimo.galli@unimi.it

[7] Department of Biomedical and Clinical Sciences, University of Milan, 20157 Milan, Italy

[8] Management of the Vaccination Campaign, Lombardy Region, 20124 Milan, Italy; bertolaso1@gmail.com

* Correspondence: giovanni.corrao@unimib.it; Tel.: +39-02-64485854

Section S1. Formal justification for choosing the number of controls to be matched with each vaccinated citizen

From a statistical standpoint, sample size calculations for Poisson-distributed data are characterized by the parameter λ, which is the number of events, divided by the number of the considered units (e.g., person-months). Sample size calculations to test the hypothesis that the true incidence rates in two groups of individuals are equal, the approach by Lwanga & Lemeshow was used [30]. As an example, consider the age-gender category in which we expect that Covid-19 related events to be less frequent, that is women under 50 years of age. Let the mean count of Covid-19 related outcome (i.e., the event which vaccine should prevent) to be 5 events per 10,000 person-months uncovered by vaccine exposure ($\lambda_0 = 0.0005$, accumulated during a given amount of person-months, PM_0). If we want to appreciate an at least one-half decreased rate during person-months covered by vaccine exposure (λ_1=0.00025, accumulated during a given amount of PM_1), with a power of 0.80 and a one-side first type error of 0.05, we should ensure that at least 94,187 person-months are accumulated by both the vaccinated cohort members and by the 1:1 matched controls.

Let consider now the harmful events possibly be due to vaccine exposure, such as the venous thromboembolic outcomes took into account in the current study. With respect to our line of reasoning, the only change is the expected frequency of the event, which in this case is expected to be rarer (say, $\lambda_0 = 0.00003$ always among the women under 50 years of age). In this case, by including 100,000 person-months we should be able of appreciating increased rate of 5-fold or more (i.e., $\lambda_1 = 0.00015$). As we were interested of appreciating a 3-fold increased rate during periods covered by vaccine exposure, we reasoned on the opportunity of increasing the number of controls to be matched to each vaccinated person for reaching the desiderate minimum appreciable

incidence rate ratio. For this reason, a 1:10 matched design was adopted for estimating vaccine safety

Table S1. List of conditions used for typifying vaccinated cohort members and unvaccinated controls.

Diagnostic categories	#	Disease / condition	ICD-9 CM	ATC
Infectious and parasitic diseases	1	HIV infection	042.x, V08	J05AB14, J05AE, J05AF01, J05AF02, J05AF04, J05AF05, J05AF06, J05AF09. J05AG, J05AR, J05AX07, J05AX08, J05AX09, J05AX12
	2	Tuberculosis and Other infectious and parasitic diseases	010.x - 018.x, 001.x-009.x, 020.x-027.x, 030.x-0.41.x, 045.x–057.x, 060.x-066.x, 070.x-088.x, 090.x-104.x, 110.x-118.x, 120.x-139.x	J04AB
Neoplasms	3	Solid malignancies and Neoplasm of lymphatic and haematopoietic tissue	140.x-165.x, 170.x-176.x, 179.x-199.x, V58.0, 92.2, 200.x-208-x	L01, L03AC, L02BA01, L02BA02, L02BG02, L02BG03, L02BG04, L02BG06, L02BB01, L02BB03, L02AE02, L02AE04, L02AB01
	4	Benign neoplasm and carcinoma in situ	210.x-234.x	
Endocrine, nutritional and metabolic diseases, and immunity disorders	5	Hypothyroidism	243, 244.x	H03A, H03B
	6	Hyper e hypoparathyroidism	252.0, 252.1	
	7	Diabetes without insulin therapy	250.x, 348.0x, 357.2, 362.0, 366.41	A10B
	8	Insulin therapy		A10A
	9	Dyslipidaemia	272.2, 272.4	C10
	10	Obesity	278.0x	
	11	Weight loss	260-263.x	
	12	Disorders of fluid, electrolyte, and acid-base balance	276.x	
	13	Gout	274.x	M04AC01, M04AA, M04AB
	14	Other disorders of endocrine, nutritional and metabolic diseases	240.x-242.x, 245.x, 246.x, 249.x, 251.x, 252.8, 252.9, 253.x-259.x, 264.x-269.x, 270.x, 271.x, 272.0, 272.1, 272.3, 272.5-272.9, 273.x, 275.x, 277.x, 278.1-278.8 (except 277.0)	
	15	Disorders involving the immune mechanisms	279.x	
Diseases of the blood and blood-forming organs	16	Coagulation defects	286.x	B02B
	17	Autoimmune haemolytic anaemias, Other anaemias, Anaemias only tracked from drug therapy	280.x-282.x, 283.1-283.9, 284.x-285.x	B03A, B03B, B03XA01, L03AA
	18	Other diseases of the blood and blood-forming organs	287.x-289.x	
Mental disorders	19	Dementia / Alzheimer	290.0-290.4x, 331.0x	N06DA, N06DX01

	20	Psychosis	295.x, 297.x, 298.2-298.9, 299.1x	N05AD, N05AA, N05AB, N05AC, N05AX, N05AE, N05AF, N05AG N05AH, N05AL
	21	Depression	296.2, 296.3, 296.82, 298.0, 300.4, 301.12, 309.0x, 309.1x, 311.x	N06A
	22	Bipolar disorders	296.0x, 296.1x, 296.4x, 296.5x, 296.6x, 296.7x, 296.80, 296.81, 296.89, 296.9x, 298.1x	N05AN
	23	Alcohol abuse	291.1, 291.2, 291.5, 291.8x, 291.9, 303.9, 305.0x, V11.3x	N07BB01
	24	Drug addition	292.0x, 292.82-292.89, 292.9x, 304.x, 305.2x-305.9x	N07BB04
	25	Anxiety	300.0x	N05BA, N05BB01, N05CD, N05BC01, N05BC51, N05BX, N05CF, N05CX01, N06BX
	26	Other mental disorders	290.8, 290.9, 291.0, 291.3, 291.4, 292.1x, 292.2, 292.81, 293.x, 294.x, 299.0x, 299.8x, 299.9x, 300.0x-300.2x, 300.3, 300.5-300.9, 301.0, 301.10, 301.11, 301.2x-301.9x, 302.x, 303.x, 305.1, 306.x-308.x, 309.2x-309.4x, 310.x, 312.x-319.x	
Diseases of the nervous system and sense organs	27	Parkinson's disease and parkinsonism	332.x	N04
	28	Multiple sclerosis	340	L03AB07, L03AB08, L04AA23, L04AA27, L03AX13, L04AA31, L04AA34, L03AB13, L04AX07
	29	Epilepsy and recurrent seizures	345.x	N03AF01, N03AB02, N03AA02, N03AA03, N03AA04, N03AE01, N03AD01, N03AG01, N05BA09, N03AG04, N03AX10, N03AG06, N03AF02, N03AX14, N03AX15
	30	Glaucoma	365.x	S01E
	31	Disorders of the eye and adnexa	360.x-379.x (except 365.x)	
	32	Diseases of the ear and mastoid process	380.x-389.x	
	33	Other diseases of the nervous system and sense organs	320.x-326.x, 330.x-331.x, 333.x-337.x, 340.x-344.x, 346.x-359.x	
Diseases of the circulatory system	34	Ischaemic Heart Disease/Angina	410.x – 414	C01DA, C01DX
	35	Heart failure	398.91, 402.11, 402.91, 404.11, 404.13, 404.91, 404.93, 428.x	
	36	Arrhythmia	426.10, 426.11, 426.13, 426.20-426.53, 426.60-426.89, 427.0, 427.2,	C01BA, C01BC, C01BD

			427.31, 427.60,427.9, 785.0x, V45.0x, V53.3x	
	37	Valvular diseases	093.20-093.24, 394.0x-397.1x, 424.00-424.91, 746.3x-746.6x, V42.2x, V43.3x	
	38	Vascular diseases	440.x, 441.2, 441.4, 441.7, 441.9, 443.1x-443.9x, 447.1, 557.1x, 557.9x, 785.4x, V43.4x	
	39	Cerebrovascular diseases	430.x-438.x	
	40	Hypertension	401.x-405.x	C03AA, C03AB, C03AH, C03AX01, C02CA04, C03BA02, C03BA03, C03BA04, C03BA05, C03BA07, C03BA08, C03BA09, C03BA10, C03BA11, C03DB01, C03DB02, C03EA, C09BA02, C09BA03, C09BA04, C09BA05, C09BA06, C09BA07, C09BA08, C09BA09, C09BB, C09DB, C09DA01, C09DA02, C09DA03, C09DA04, C09DA06, C09DA07, C09DA08, C02AB01, C02AB02, C02AC01, C02AC02, C02AC04, C02AC05, C02DB02, C02DB03, C02DB04, C02DC01, C02DD01, C02DG01, C02KA01, C02KB01, C02KC01, C02KD01, C02KX01, C09XA
	41	Coronary and peripheral vascular disease		B01AB, B01AX01, B01AD10, B01AD12, C04AD03, B01AC05
	42	Oral anticoagulant agents		B01AA, B01AE, B01AF
	43	Other diseases of the circulatory system	390.x-392.x, 393, 397.9, 398.90, 398.99, 411.8x, 412.x-417x, 420.x-423.x, 424.99, 425.x, 426.0, 426.12, 426.54, 426.9, 427.1, 427.32, 427.4x, 427.5, 427.61, 427.69, 427.8x, 429.x, 441.0x, 441.1, 441.3, 441.5, 441.6, 442.x, 443.0, 444.x-446.x, 447.0, 447.2-447.9, 448.x 451.x-459.x	
Diseases of the respiratory system	44	Chronic Obstructive Pulmonary Disease, Asthma, Chronic respiratory disease only tracked from drug therapy	490-492.x, 493.x, 494.x, 496	R03AA, R03AB, R03AC, R03DA, R03DB, R03DA20, R01AC01, R03BC01, R01AC51, S01GX01, S01GX51, R03BA

	45	Acute respiratory infections	460-466.x	
	46	Cystic Fibrosis	277.0	R05CB, R05FB01, R05FA01, A09AA02, R07AX02, R07AX30, R07AX31
	47	Other diseases of the respiratory system	470.x-478.x, 480.x-487.x, 495.x, 500.x-508.x, 510.x-519.x	
Diseases of the digestive system	48	Liver cirrhosis and other liver chronic diseases	571.x, 573.x	J05AP08, J05AP09, J05AP51, J05AP53, J05AP54, J05AP55, J05AP56, J05AP57, B05AA01
	49	Inflammatory bowel diseases (Ulcerative colitis and Chron's disease)	555.x-556.x	A07EC01, A07EC02, A07EC03, A07EC04
	50	Chronic and acute pancreatitis	577.0-577.1	
	51	Other diseases of the digestive system	520.x-553.x, 557.x-570, 572.x, 574.x-576.x, 577.2-577.9, 578.x, 579.x	
Diseases of the genitourinary system	52	Chronic kidney disease	585, V45.1, V56.x, V03AE	
	53	Other kidney disorders	580.x-584.x, 586, 587, 588.x-589.x	
	54	Other diseases of the genitourinary system	590.x-608.x, 610.x, 611.x, 614.x-629.x	
Diseases of the skin and subcutaneous tissues	55	Diseases of the skin and subcutaneous tissues, including No rheumatoid psoriasis	680.x-686.x, 690.x-695.x, 696.0, 696.2-696.5, 696.8, 697.x, 698.x, 700.x-709.x, 696.1	D05BB01, D05BB02, D05AX
Diseases of the musculoskeletal system and connective tissue	56	Autoimmune disease (Rheumatoid arthritis, Rheumatoid psoriasis, Anchylosing spondylitis, Systemic sclerosis, Systemic lupus erythematosus)	714.0, 696.0, 720.0, 710.1x, 710.0x	
	57	Other diseases of the musculoskeletal system and connective tissue	710.2-710.9, 711.x-713.x, 714.1x, 714.9x, 715.x-719.x, 720.1x-720.9x, 721.x-739.x	
Symptoms, signs and ill-defined conditions	58	Symptoms, signs and ill-defined conditions	780-799	
Other conditions	59	Transplantation	V42	L04AA01, L04AA02, L04AA03, L04AA04, L04AA05, L04AA06, L04AA08, L04AA09, L04AA10, L04AA11, L04AA12, L04AA14, L04AA15, L04AA16, L04AA17, L04AA18, L04AA19, L04AA21, L04AD01, L04AD02, L04AX01

60	Chronic pain	338.2, 338.4	N02AA01, N02AG01, N02AE01, N02AB03, N02AA05, N02AA55, N02AA03, N02AX06
61	Corticosteroids		H02
62	Hormone therapy (oral contraceptives or replacement hormone therapy)		G03AA, G03AB, G02BB01, G03HB01, G03CA

Table S2. ICD-9 codes used for tracking venous thromboembolic outcome events.

ICD-9 codes	Diagnosis
415.1	Pulmonary embolism and infarction
437.6	Nonpyogenic thrombosis of intracranial venous system
451	Deep vein thrombosis
452	Portal vein obstruction
453.0	Budd-Chiari syndrome (hepatic vein thrombosis)
453.1	Thrombophlebitis migrans
453.2	Embolism of Vena Cava
453.3	Embolism and thrombosis in renal vein
557.1	Mesenteric vein thrombosis

Table S3. Benefit profile according with vaccine type, gender and age category obtained by changing the time-window length after vaccine inoculation assumed of not involving vaccination protection because immunity is gradually building. Time-windows of 7 and 21 days were considered for this secondary analysis alternative to 14 days of the main analysis.

Oxford-AstraZeneca, women, 7 days

Age category (years)	Exposure to vaccine benefits (first dose)			Unexposure to vaccine benefits			Incidence rate ratio (95% CI) [(a)]		Number needed to treat (95% CI) [(b)]
	# events	# PM	IR (per 10,000 PM)	# events	# PM	IR (per 10,000 PM)	Unadjusted	Adjusted	
< 50	15	220,308	0.68	103	277,919	3.71	0.18 (0.11-0.32)	0.19 (0.11-0.32)	3,331 (402-4,535)
50-59	13	108,298	1.20	73	136,288	5.36	0.22 (0.13-0.41)	0.23 (0.13-0.42)	2,406 (293-3,622)
60-69	24	67,347	3.56	88	102,880	8.55	0.42 (0.27-0.67)	0.48 (0.31-0.77)	2,004 (478-3,792)
70-79	22	85,486	2.57	178	163,370	10.90	0.24 (0.16-0.39)	0.28 (0.18-0.43)	1,202 (333-1,578)
≥ 80	3	2,563	11.70	6	3,806	15.77	0.74 (0.19-2.97)	0.86 (0.20-3.74)	-
Total	77	484,003	1.59	448	684,263	6.55			

Oxford-AstraZeneca, women, 21 days

Age category (years)	Exposure to vaccine benefits (first dose)			Unexposure to vaccine benefits			Incidence rate ratio (95% CI) [(a)]		Number needed to treat (95% CI) [(b)]
	# events	# PM	IR (per 10,000 PM)	# events	# PM	IR (per 10,000 PM)	Unadjusted	Adjusted	
< 50	6	161,151	0.37	113	323,010	3.50	0.11 (0.05-0.24)	0.11 (0.05-0.25)	3,212 (170-4,165)
50-59	5	80,337	0.62	81	160,759	5.04	0.12 (0.05-0.31)	0.13 (0.05-0.32)	2,264 (121-3,140)
60-69	8	39,843	2.01	104	130,068	8.00	0.25 (0.12-0.53)	0.29 (0.14-0.59)	1,670 (173-2,602)
70-79	4	39,459	1.01	196	209,391	9.36	0.11 (0.04-0.30)	0.12 (0.05-0.34)	1,198 (73-1,499)
≥ 80	1	1,474	6.78	8	4,897	16.34	0.42 (0.05-3.32)	0.51 (0.06-4.23)	-
Total	24	322,264	0.74	502	828,125	6.06			

Oxford-AstraZeneca, men, 7 days

Age category (years)	Exposure to vaccine benefits (first dose)			Unexposure to vaccine benefits			Incidence rate ratio (95% CI) [a]		Number needed to treat (95% CI) [b]
	# events	# PM	IR (per 10,000 PM)	# events	# PM	IR (per 10,000 PM)	Unadjusted	Adjusted	
< 50	19	118,340	1.61	66	148,358	4.45	0.36 (0.22-0.62)	0.38 (0.22-0.63)	3,517 (516-6,498)
50-59	16	55,041	2.91	82	69,090	11.87	0.24 (0.14-0.42)	0.25 (0.15-0.43)	1,116 (222-1,662)
60-69	18	49,870	3.61	103	78,297	13.15	0.27 (0.17-0.46)	0.29 (0.18-0.49)	1,048 (241-1,543)
70-79	64	67,048	9.55	254	129,963	19.54	0.49 (0.40-0.69)	0.57 (0.43-0.75)	1,000 (537-1,536)
≥ 80	4	1,635	24.47	8	2,415	33.13	0.74 (0.22-2.45)	0.91 (0.25-3.36)	-
Total	121	291,934	4.14	513	428,123	11.98	0.36 (0.22-0.62)	0.38 (0.22-0.63)	

Oxford-AstraZeneca, men, 21 days

Age category (years)	Exposure to vaccine benefits (first dose)			Unexposure to vaccine benefits			Incidence rate ratio (95% CI) [a]		Number needed to treat (95% CI) [b]
	# events	# PM	IR (per 10,000 PM)	# events	# PM	IR (per 10,000 PM)	Unadjusted	Adjusted	
< 50	4	83,403	0.48	82	169,773	4.83	0.10 (0.04-0.28)	0.10 (0.04-0.28)	2,299 (94-3,122)
50-59	3	39,834	0.75	95	80,064	11.87	0.06 (0.01-0.15)	0.06 (0.02-0.20)	900 (41-1,165)
60-69	3	28,568	1.05	118	99,255	11.89	0.09 (0.03-0.28)	0.09 (0.03-0.30)	923 (45-1,193)
70-79	2	30,652	0.65	316	166,360	19.00	0.03 (0.02-0.20)	0.04 (0.01-0.15)	545 (26-624)
≥ 80	1	945	10.58	11	3,105	35.43	0.30 (0.04-2.31)	0.34 (0.04-2.71)	-
Total	13	183,401	0.71	622	518,556	12.00			

Pfizer/Moderna, women, 7 days

Age category (years)	Exposure to vaccine benefits (first dose)			Unexposure to vaccine benefits			Incidence rate ratio (95% CI) [a]		Number needed to treat (95% CI) [b]
	# events	# PM	IR (per 10,000 PM)	# events	# PM	IR (per 10,000 PM)	Unadjusted	Adjusted	
< 50	28	107,018	2.62	179	458,690	3.90	0.67 (0.45-1.00)	0.58 (0.39-0.88)	6,101 (3,695-17,494)
50-59	24	74,243	3.23	161	296,328	5.43	0.59 (0.39-0.91)	0.51 (0.33-0.79)	3,756 (2,425-8,333)
60-69	22	60,101	3.66	164	182,173	9.00	0.41 (0.27-0.65)	0.38 (0.24-0.59)	1,792 (1,315-2,809)
70-79	54	55,991	9.64	198	142,035	13.94	0.69 (0.55-1.01)	0.65 (0.48-0.88)	2,050 (1,244-5,818)
≥ 80	476	163,875	29.05	2007	517,441	38.79	0.75 (0.71-0.86)	0.77 (0.69-0.85)	1,121 (829-1,731)
Total	604	461,227	13.10	2709	1,596,666	16.97			

Pfizer/Moderna, women, 21 days

Age category (years)	Exposure to vaccine benefits (first dose)			Unexposure to vaccine benefits			Incidence rate ratio (95% CI) [a]		Number needed to treat (95% CI) [b]
	# events	# PM	IR (per 10,000 PM)	# events	# PM	IR (per 10,000 PM)	Unadjusted	Adjusted	
< 50	5	86,941	0.58	206	515,798	3.99	0.13 (0.06-0.35)	0.13 (0.05-0.32)	2,878 (2,381-3,638)
50-59	0	57,036	0.00	187	336,276	5.56	-	-	-
60-69	1	34,242	0.29	186	216,700	8.58	0.03 (0.00-0.25)	0.03 (0.00-0.23)	1,206 (1,036-1,443)
70-79	7	24,864	2.82	248	172,794	14.35	0.19 (0.10-0.44)	0.19 (0.09-0.39)	860 (697-1,123)
≥ 80	91	120,342	7.56	2413	622,229	38.78	0.19 (0.16-0.25)	0.19 (0.16-0.25)	320 (299-345)
Total	104	323,425	3.26	3240	1,863,797	17.39			

Pfizer/Moderna, men, 7 days

Age category (years)	Exposure to vaccine benefits (first dose)			Unexposure to vaccine benefits			Incidence rate ratio (95% CI) [a]		Number needed to treat (95% CI) [b]
	# events	# PM	IR (per 10,000 PM)	# events	# PM	IR (per 10,000 PM)	Unadjusted	Adjusted	
< 50	19	66,697	2.85	86	252,711	3.40	0.84 (0.53-1.45)	0.70 (0.42-1.18)	-
50-59	25	47,873	5.22	148	157,073	9.42	0.55 (0.38-0.88)	0.46 (0.30-0.72)	1,965 (1,335-3,727)
60-69	69	56,434	12.23	265	168,445	15.73	0.78 (0.60-1.03)	0.68 (0.52-0.89)	1,986 (1,200-5,756)
70-79	86	52,322	16.44	340	140,313	24.23	0.68 (0.56-0.91)	0.64 (0.50-0.81)	1,146 (771-2,233)
≥ 80	375	89,637	41.84	1641	289,685	56.65	0.74 (0.69-0.87)	0.75 (0.67-0.84)	706 (520-1,100)
Total	574	312,963	18.34	2480	1,008,227	24.60			

Pfizer/Moderna, men, 21 days

Age category (years)	Exposure to vaccine benefits (first dose)			Unexposure to vaccine benefits			Incidence rate ratio (95% CI) [a]		Number needed to treat (95% CI) [b]
	# events	# PM	IR (per 10,000 PM)	# events	# PM	IR (per 10,000 PM)	Unadjusted	Adjusted	
< 50	6	49,231	1.22	100	286,251	3.49	0.35 (0.16-0.83)	0.30 (0.13-0.69)	4,396 (2,885-9,232)
50-59	4	32,164	1.24	171	181,239	9.44	0.13 (0.05-0.37)	0.11 (0.04-0.31)	1,221 (994-1,581)
60-69	5	31,520	1.59	332	200,617	16.55	0.10 (0.04-0.23)	0.09 (0.04-0.21)	668 (581-787)
70-79	11	24,533	4.48	418	170,165	24.56	0.18 (0.10-0.35)	0.18 (0.10-0.32)	498 (423-605)
≥ 80	55	65,238	8.43	1980	352,287	56.20	0.15 (0.12-0.20)	0.15 (0.12-0.20)	209 (196-225)
Total	81	202,687	4.00	3001	1,190,559	25.21			

Table S4. Harm profile according with vaccine type, gender and age category obtained by changing the time-window length at risk of developing venous thromboembolism. Time-windows of 21 and 35 days were considered for this secondary analysis alternative to 28 days of the main analysis.

Oxford-AstraZeneca, women, 21 days

Age category (years)	Exposure to vaccine benefits (first dose)			Unexposure to vaccine benefits			Incidence rate ratio (95% CI) [a]		Number needed to harm (95% CI) [b]
	# events	# PM	IR (per 10,000 PM)	# events	# PM	IR (per 10,000 PM)	Unadjusted	Adjusted	
< 50	6	75,027	0.80	22	750,270	0.29	2.73 (1.11-6.73)	2.90 (1.15-7.31)	17,949 (8,142-87,716)
50-59	4	38,835	1.03	18	388,336	0.46	2.22 (0.75-6.57)	2.48 (0.83-7.38)	-
60-69	3	44,947	0.67	43	449,395	0.96	0.70 (0.22-2.25)	0.73 (0.22-2.37)	-
70-79	9	84,832	1.06	263	847,777	3.10	0.34 (0.15-0.61)	0.32 (0.16-0.64)	-
≥ 80	0	1,703	0.00	9	16,950	5.31	-	-	-
Total	22	245,343	0.90	355	2,452,728	1.45			

Oxford-AstraZeneca, women, 35 days

Age category (years)	Exposure to vaccine benefits (first dose)			Unexposure to vaccine benefits			Incidence rate ratio (95% CI) [a]		Number needed to harm (95% CI) [b]
	# events	# PM	IR (per 10,000 PM)	# events	# PM	IR (per 10,000 PM)	Unadjusted	Adjusted	
< 50	7	123,838	0.57	35	1,238,389	0.28	2.00 (0.89-4.50)	2.12 (0.93-4.85)	-
50-59	4	63,700	0.63	38	636,955	0.60	1.05 (0.38-2.95)	1.20 (0.43-3.38)	-
60-69	5	65,352	0.77	64	653,353	0.98	0.78 (0.31-1.94)	0.87 (0.35-2.17)	-
70-79	13	112,687	1.15	312	1,125,687	2.77	0.42 (0.22-0.68)	0.40 (0.23-0.72)	-
≥ 80	0	2,631	0.00	17	26,134	6.50	-	-	-
Total	29	368,208	0.79	466	3,680,518	1.27			

Oxford-AstraZeneca, men, 21 days

Age category (years)	Exposure to vaccine benefits (first dose)			Unexposure to vaccine benefits			Incidence rate ratio (95% CI) [a]		Number needed to harm (95% CI) [b]
	# events	# PM	IR (per 10,000 PM)	# events	# PM	IR (per 10,000 PM)	Unadjusted	Adjusted	
< 50	1	38,108	0.26	29	381,067	0.76	0.34 (0.05-2.53)	0.34 (0.05-2.49)	-
50-59	2	18,535	1.08	24	185,340	1.29	0.83 (0.20-3.53)	0.86 (0.20-3.66)	-
60-69	8	35,248	2.27	81	352,399	2.30	0.99 (0.48-2.04)	0.98 (0.47-2.04)	-
70-79	11	67,952	1.62	289	678,568	4.26	0.38 (0.21-0.69)	0.36 (0.20-0.67)	-
≥ 80	1	1,067	9.37	9	10,599	8.49	1.10 (0.14-8.71)	1.25 (0.15-10.65)	-
Total	23	160,910	1.43	432	1,607,972	2.69			

Oxford-AstraZeneca, men, 35 days

Age category (years)	Exposure to vaccine benefits (first dose)			Unexposure to vaccine benefits			Incidence rate ratio (95% CI) [a]		Number needed to harm (95% CI) [b]
	# events	# PM	IR (per 10,000 PM)	# events	# PM	IR (per 10,000 PM)	Unadjusted	Adjusted	
< 50	1	63,129	0.16	38	631,260	0.60	0.26 (0.04-1.92)	0.25 (0.03-1.80)	-
50-59	3	30,563	0.98	30	305,612	0.98	1.00 (0.31-3.28)	1.03 (0.31-3.40)	-
60-69	11	50,509	2.18	112	504,904	2.22	0.98 (0.53-1.82)	0.99 (0.53-1.86)	-
70-79	17	89,529	1.90	362	893,234	4.05	0.47 (0.29-0.76)	0.45 (0.27-0.73)	-
≥ 80	1	1,655	6.04	10	16,393	6.10	0.99 (0.13-7.74)	1.18 (0.14-9.90)	-
Total	33	235,386	1.40	552	2,351,403	2.35			

Pfizer/Moderna, women, 21 days

Age category (years)	Exposure to vaccine benefits (first dose)			Unexposure to vaccine benefits			Incidence rate ratio (95% CI) [a]		Number needed to harm (95% CI) [b]
	# events	# PM	IR (per 10,000 PM)	# events	# PM	IR (per 10,000 PM)	Unadjusted	Adjusted	
< 50	9	99,037	0.91	27	986,336	0.27	3.32 (1.56-7.06)	2.09 (0.94-4.64)	-
50-59	0	69,543	0.00	24	692,689	0.35	-	-	-
60-69	9	67,897	1.33	75	676,581	1.11	1.20 (0.60-2.39)	0.86 (0.43-1.74)	-
70-79	15	69,950	2.14	242	697,201	3.47	0.62 (0.34-0.99)	0.47 (0.27-0.80)	-
≥ 80	75	215,753	3.48	1,114	2,099,026	5.31	0.65 (0.52-0.83)	0.66 (0.52-0.83)	-
Total	108	522,180	2.07	1,482	5,151,833	2.88			

Pfizer/Moderna, women, 35 days

Age category (years)	Exposure to vaccine benefits (first dose)			Unexposure to vaccine benefits			Incidence rate ratio (95% CI) [a]		Number needed to harm (95% CI) [b]
	# events	# PM	IR (per 10,000 PM)	# events	# PM	IR (per 10,000 PM)	Unadjusted	Adjusted	
< 50	11	161,757	0.68	48	1,610,972	0.30	2.28 (1.19-4.39)	1.39 (0.70-2.78)	-
50-59	2	111,832	0.18	48	1,113,894	0.43	0.42 (0.10-1.71)	0.29 (0.07-1.21)	-
60-69	11	98,126	1.12	102	977,919	1.04	1.07 (0.58-2.00)	0.79 (0.42-1.48)	-
70-79	19	98,382	1.93	293	980,748	2.99	0.65 (0.38-0.99)	0.51 (0.31-0.82)	-
≥ 80	113	313,134	3.61	1,602	3,036,236	5.28	0.68 (0.57-0.83)	0.68 (0.56-0.83)	-
Total	156	783,231	1.99	2,093	7,719,769	2.71			

Pfizer/Moderna, men, 21 days

Age category (years)	Exposure to vaccine benefits (first dose)			Unexposure to vaccine benefits			Incidence rate ratio (95% CI) [(a)]		Number needed to harm (95% CI) [(b)]
	# events	# PM	IR (per 10,000 PM)	# events	# PM	IR (per 10,000 PM)	Unadjusted	Adjusted	
< 50	1	61,000	0.16	15	607,180	0.25	0.66 (0.09-5.02)	0.69 (0.09-5.24)	-
50-59	11	44,220	2.49	33	439,986	0.75	3.32 (1.68-6.56)	2.32 (0.97-5.53)	-
60-69	19	64,393	2.95	152	641,386	2.37	1.25 (0.77-2.01)	0.87 (0.53-1.42)	-
70-79	20	66,672	3.00	274	664,420	4.12	0.73 (0.46-1.15)	0.64 (0.40-1.01)	-
≥ 80	45	129,497	3.48	850	1,252,558	6.79	0.51 (0.37-0.68)	0.50 (0.37-0.68)	-
Total	96	365,781	2.62	1,324	3,605,530	3.67			

Pfizer/Moderna, men, 35 days

Age category (years)	Exposure to vaccine benefits (first dose)			Unexposure to vaccine benefits			Incidence rate ratio (95% CI) [(a)]		Number needed to harm (95% CI) [(b)]
	# events	# PM	IR (per 10,000 PM)	# events	# PM	IR (per 10,000 PM)	Unadjusted	Adjusted	
< 50	3	99,109	0.30	24	986,448	0.24	1.24 (0.37-4.13)	0.73 (0.21-2.61)	-
50-59	12	70,288	1.71	50	699,322	0.72	2.39 (1.27-4.48)	1.59 (0.80-3.16)	-
60-69	26	92,779	2.80	196	924,262	2.12	1.32 (0.88-1.99)	0.95 (0.63-1.45)	-
70-79	23	93,631	2.46	357	933,047	3.83	0.64 (0.42-0.98)	0.57 (0.37-0.87)	-
≥ 80	67	183,442	3.65	1,148	1,763,492	6.51	0.56 (0.43-0.71)	0.55 (0.43-0.70)	-
Total	131	539,249	2.43	1,775	5,306,570	3.34			

Table S5. Benefit –harm profiles according with vaccine type, gender and age category obtained by propensity score adjustment.

Oxford-AstraZeneca, Women

Age category (years)	Exposure to vaccine benefits (first dose)			Unexposure to vaccine benefits			Adjusted incidence rate ratio (95% CI)	Number needed to treat (95% CI) [b]
	# events	# PM	IR (per 10,000 PM)	# events	# PM	IR (per 10,000 PM)		
< 50	7	198,035	0.35	111	302,575	3.67	0.10 (0.05 to 0.22)	3,029 (191-3,893)
50-59	8	95,952	0.83	78	149,102	5.23	0.16 (0.08 to 0.34)	2,274 (180-3,226)
60-69	12	52,563	2.28	100	117,698	8.50	0.30 (0.16 to 0.55)	1,609 (234-2,463)
70-79	10	58,529	1.71	190	190,324	9.98	0.21 (0.11 to 0.39)	1,209 (184-1,555)
≥ 80	1	1,997	5.01	8	4,373	18.29	0.27 (0.03 to 2.18)	-
Total	38	407,077	0.93	487	764,072	6.37		

Age category (years)	Exposure to vaccine harms (first dose)			Unexposure to vaccine harms			Adjusted incidence rate ratio (95% CI)	Number needed to harm (95% CI) [b]
	# events	# PM	IR (per 10,000 PM)	# events	# PM	IR (per 10,000 PM)		
< 50	7	99,556	0.70	30	995,563	0.30	2.74 (1.19-6.28)	19,072 (9,099-198,691)
50-59	4	51,391	0.78	28	513,884	0.54	1.53 (0.54-4.39)	-
60-69	4	56,267	0.71	53	562,560	0.94	0.82 (0.29-2.26)	-
70-79	12	100,237	1.20	293	1,001,500	2.93	0.43 (0.24-0.79)	-
≥ 80	0	2,191	0.00	12	21,792	5.51	-	-
Total	27	309,642	0.87	416	3,095,299	1.34		

Oxford-AstraZeneca, Men

Age category (years)	Exposure to vaccine benefits (first dose)			Unexposure to vaccine benefits			Adjusted incidence rate ratio (95% CI)	Number needed to treat (95% CI) [b]
	# events	# PM	IR (per 10,000 PM)	# events	# PM	IR (per 10,000 PM)		
< 50	11	107,608	1.02	74	160,921	4.60	0.21 (0.11 to 0.39)	2,796 (255-4,210)
50-59	8	49,469	1.62	90	75,166	11.97	0.14 (0.07 to 0.28)	966 (116-1,310)
60-69	9	38,341	2.35	112	89,850	12.47	0.20 (0.10 to 0.40)	988 (133-1,366)
70-79	22	45,579	4.83	296	151,433	19.55	0.28 (0.18 to 0.44)	679 (222-860)
≥ 80	2	1,277	15.66	10	2,772	36.08	0.44 (0.10 to 2.02)	-
Total	52	242,274	2.15	582	480,142	12.12		

Age category (years)	Exposure to vaccine harms (first dose)			Unexposure to vaccine harms			Adjusted incidence rate ratio (95% CI)	Number needed to harm (95% CI) [b]
	# events	# PM	IR (per 10,000 PM)	# events	# PM	IR (per 10,000 PM)		
< 50	1	50,669	0.20	33	506,677	0.65	0.33 (0.04-2.39)	-
50-59	3	24,608	1.22	28	246,071	1.14	1.10 (0.33-3.62)	-
60-69	8	43,846	1.82	98	438,323	2.26	0.90 (0.44-1.86)	-
70-79	14	79,934	1.75	332	797,823	4.16	0.48 (0.28-0.83)	-
≥ 80	1	1,374	7.28	10	13,623	7.34	1.00 (0.13-7.84)	-
Total	27	200,431	1.35	501	2,002,517	2.50		

Pfizer/Moderna, Women

Age category (years)	Exposure to vaccine benefits (first dose)			Unexposure to vaccine benefits			Adjusted incidence rate ratio (95% CI)	Number needed to treat (95% CI) [b]
	# events	# PM	IR (per 10,000 PM)	# events	# PM	IR (per 10,000 PM)		
< 50	13	100,665	1.29	196	490,974	3.99	0.11 (0.02 to 0.82)	2,815 (2,355-3,496)
50-59	6	67,909	0.88	180	318,755	5.65	0.08 (0.01 to 0.60)	1,925 (1,623-2,365)
60-69	9	48,052	1.87	178	202,892	8.77	0.25 (0.09 to 0.66)	1,520 (1,187-2,113)
70-79	20	45,489	4.40	234	164,078	14.26	0.15 (0.07 to 0.33)	825 (695-1,015)
≥ 80	248	142,079	17.46	2,248	572,002	39.30	0.18 (0.04 to 0.71)	310 (291-332)
Total	296	404,194	7.32	3,036	1,748,702	17,36		

Age category (years)	Exposure to vaccine harms (first dose)			Unexposure to vaccine harms			Adjusted incidence rate ratio (95% CI)	Number needed to harm (95% CI) [b]
	# events	# PM	IR (per 10,000 PM)	# events	# PM	IR (per 10,000 PM)		
< 50	10	131,525	0.76	38	1,309,922	0.29	2.23 (1.09-4.57)	28,026 (12,482-114,225)
50-59	2	91,961	0.22	34	916,009	0.37	0.49 (0.12-2.10)	-
60-69	10	84,627	1.18	93	843,316	1.10	0.94 (0.49-1.82)	-
70-79	16	85,737	1.89	273	854,630	3.19	0.50 (0.30-0.84)	-
≥ 80	99	268,027	3.69	1385	2,603,444	5.32	0.69 (0.56-0.84)	-
Total	137	661,878	2.07	1823	6,527,321	2.79		

Pfizer/Moderna, Men

Age category (years)	Exposure to vaccine benefits (first dose)			Unexposure to vaccine benefits			Adjusted incidence rate ratio (95% CI)	Number needed to treat (95% CI) [(b)]
	# events	# PM	IR (per 10,000 PM)	# events	# PM	IR (per 10,000 PM)		
< 50	8	61,389	1.30	98	272,808	3.59	0.38 (0.05 to 2.83)	-
50-59	9	42,272	2.13	166	171,452	9.68	0.13 (0.02 to 0.93)	1,187 (976-1,514)
60-69	21	44,774	4.69	315	187,785	16.77	0.04 (0.01 to 0.30)	621 (552-709)
70-79	36	42,957	8.38	393	161,173	24.38	0.16 (0.08 to 0.30)	488 (425-574)
≥ 80	179	76,801	23.31	1844	321,868	57.29	0.20 (0.05 to 0.81)	218 (203-236)
Total	253	268,193		2816	1,115,086			

Age category (years)	Exposure to vaccine harms (first dose)			Unexposure to vaccine harms			Adjusted incidence rate ratio (95% CI)	Number needed to harm (95% CI) [(b)]
	# events	# PM	IR (per 10,000 PM)	# events	# PM	IR (per 10,000 PM)		
< 50	1	81,069	0.12	18	806,954	0.22	0.48 (0.06-3.79)	-
50-59	12	58,442	2.05	47	581,519	0.81	2.21 (0.95-5.13)	-
60-69	23	80,154	2.87	175	798,403	2.19	1.06 (0.68-1.66)	-
70-79	22	81,567	2.67	322	812,848	3.96	0.61 (0.39-0.94)	-
≥ 80	58	158,754	3.65	1025	1,530,971	6.70	0.52 (0.40-0.68)	-
Total	116	459,986	2.52	1587	4,530,695	3.50		
